# Five-year sustainability of a de-implementation strategy to reduce inappropriate use of catheters: a multicentre, mixed-methods study

**DOI:** 10.1016/j.eclinm.2024.102785

**Published:** 2024-08-16

**Authors:** Tessa M.Z.X.K. van Horrik, Eva W. Verkerk, Suzanne E. Geerlings, Rudolf B. Kool, Bart J. Laan

**Affiliations:** a Amsterdam UMC, University of Amsterdam, Department of Internal Medicine-Infectious Diseases, Amsterdam Institute for Infection and Immunity, Amsterdam Public Health, the Netherlands; b Radboud University Medical Center, IQ Health Science Department, PO Box 9101, Nijmegen 6500 HB, the Netherlands

**Keywords:** De-implementation, Sustainability, Healthcare-associated infections, Low-value care

## Abstract

**Background:**

The use of peripheral intravenous catheters (PIVCs) contributes to healthcare-associated infections. In 2017, we implemented a multifaceted de-implementation strategy that successfully reduced the inappropriate use of catheters in seven hospitals in the Netherlands (RICAT-1 study). Five years later, we investigated the sustainability of this strategy and the contributing factors.

**Methods:**

Multicentre mixed-methods study (RICAT-2), consisting of an observational study and interviews in five hospitals in the Netherlands from May 2022 to June 2023. We screened adult patients with PIVCs admitted to internal medicine and non-surgical subspecialty wards. We excluded patients admitted for an elective short stay or terminally ill. Primary endpoint was the percentage of inappropriate PIVCs. We used logistic regression analyses to compare RICAT-2 to the RICAT-1 baseline data. We interviewed 18 healthcare professionals and managers involved in RICAT-1 and/or quality management. We combined thematic inductive analysis and framework analysis.

**Findings:**

In RICAT-1 baseline, we included 22.0% (282/1284) inappropriate PIVCs. In RICAT-2, we included 13.8% (154/1113) inappropriate PIVCs (odds ratio 0.76, 95% CI 0.68 to 0.84, p < 0.001). We observed no association between the number of maintained strategy components and the sustained effect. For most hospitals, a small temporary investment in a de-implementation strategy was sufficient to achieve sustained effects. The main facilitator for reducing inappropriate catheters was intrinsic motivation to reduce catheter-associated infections. Main barriers were other priorities, lack of time, and not having a dedicated clinical champion.

**Interpretation:**

Since inappropriate PIVC use was still lower after five years than before the de-implementation strategy, healthcare professionals should be encouraged to adopt this strategy.

**Funding:**

This project was funded by The 10.13039/501100001826Netherlands Organisation for Health Research and Development (project number: 839205002).

**Research in context**

**Evidence before this study**

Inappropriate use of intravenous and urinary catheters contributes to healthcare-associated infections and does not benefit patients. Therefore, this inappropriate use is considered low-value care. In 2017, we reported the results of a de-implementation strategy that successfully reduced the inappropriate use of peripheral intravenous catheters in seven hospitals in the Netherlands. To study sustainable de-implementation strategies, we searched PubMed, MEDLINE, the Cochrane Library, ClinicalTrials.gov until April 23, 2024, combining search terms “Program evaluation”, “Program Sustainability”, “Sustainability”, “Program Effectiveness”, “Quality Improvement”, “Long-term effect”, “De-implementation”, “Intervention”, “Strategy”, and “Low-value care”.

The searches resulted in over 300 publications reporting implementation and quality improvement studies. However, few studies reported sustainability of a strategy aimed at reducing low-value care and their follow-up periods were not longer than 12 months after the strategy. In addition, no study evaluated the elements of a de-implementation strategy required to achieve a sustained effect. Therefore, we developed a mixed-methods study to investigate the sustainability of a multifaceted de-implementation strategy to reduce inappropriate use of catheters five years after the de-implementation strategy was performed.

**Added value of this study**

In this mixed-methods study, we measured the percentages of inappropriate use of peripheral intravenous catheters and urinary catheters in non-surgical patients, five years after the introduction of a de-implementation strategy. Regarding inappropriate use of peripheral intravenous catheters, we found that the effect of the de-implementation strategy was sustained for at least five years. Further, we held semi-structured interviews with healthcare professionals to identify barriers and facilitators related to maintaining the strategy. Some hospitals maintained the strategy whereas others did not, and we observed no association between the sustainment of the strategy and the sustainment of the effect. The main facilitator for reducing inappropriate catheters was intrinsic motivation to reduce catheter-associated infections. The main barriers included other priorities, a lack of time, and no dedicated clinical leader.

**Implications of all the available evidence**

The effect of the multifaceted de-implementation strategy was sustained for peripheral intravenous catheters in five hospitals, regardless of whether the strategy components were actively maintained or not. The results of this study should encourage healthcare professionals to adopt this de-implementation strategy to reduce inappropriate use of catheters in clinical practice.

## Introduction

Healthcare professionals and researchers are continuously performing studies to develop and implement changes that improve the quality of care. However, few studies investigated whether the effects of de-implementation are sustained over the long term and when they do, the effects are evaluated after a relative short period.^1^, ^2^, ^3^ For example, Mafi and colleagues evaluated the long-term effects of a multifaceted de-implementation strategy that successfully reduced low-value preoperative care after already 12 months.^4^

Furthermore, it is unclear which elements of a de-implementation strategy are required to maintain an effect and what factors facilitate or impede the upkeep of the strategy after the initial study ends.^5^^,^^6^ This information is crucial in order to design sustainable de-implementation strategies. Therefore, we studied the long-term effects of a de-implementation strategy that was used in seven hospitals in the Netherlands in 2017. This multifaceted strategy consisted among other things of audit, education and competitive feedback on the number of appropriate catheters, and appointing a local champion. This strategy successfully reduced the use of inappropriate peripheral intravenous and urinary catheters in seven hospitals in the Netherlands during a seven-month period (RICAT-1 study).^7^

Five years later, we combined quantitative measurements with qualitative interviews to measure the current inappropriate catheter use. We performed a post-hoc analysis of RICAT-1 and aimed to identify whether and how the strategy and the effects were maintained, what barriers and facilitators influenced sustainability of the de-implementation strategy and the sustained reduction of inappropriate catheter use. We hypothesized that the reduction of inappropriate catheter use was maintained, even after the de-implementation efforts of the research team had stopped.

## Methods

### Study design and setting

We performed a repeated measurement of the inappropriate use of catheters, by conducting a mixed-methods study (RICAT-2) in five of the seven participating hospitals of the original RICAT-1 study.^7^ The quantitative part consisted of a multicenter observational study in the internal medicine and non-surgical subspecialty wards from May 2022 until January 2023. The qualitative part included semi-structured interviews with clinicians and managers in these wards from April 2023 until June 2023. We evaluated the strategy components of the initial de-implementation strategy that was used in RICAT-1. The following strategy components were included in the de-implementation strategy during the RICAT-1 study: audit and feedback on the number of inappropriate catheters, educational meetings for healthcare professionals, local champion, pocket cards, posters, protocols, smart phrases in the electronic health record, and nurse empowerment in catheter care.

The five included hospitals were two sites of one academic hospital, two general teaching hospitals, and one non-teaching hospital in the Netherlands. The same internal medicine and non-surgical subspecialty wards from RICAT-1 participated, except for two wards of the academic hospital, because they merged with wards that did not participate in RICAT-1. Two hospitals from the first study (hospital 2 and hospital 5 in RICAT-1) were excluded due to major organizational changes in the hospitals and limited time and budget to participate in the RICAT-2 study.

For the interpretation and synthesis of the quantitative and qualitative part, we referred to the results of RICAT-1 post-intervention measurement.

This manuscript was written in line with the Revised Standards for Quality Improvement Reporting Excellence (SQUIRE 2.0) and the Consolidated Criteria for Reporting Qualitative Research (COREQ).^8^^,^^9^

### Quantitative part

#### Patient screening and inclusion

The methods of the RICAT-2 study were similar to those of the RICAT-1 study.^7^ All adult patients admitted to the wards of internal medicine, gastroenterology, geriatrics, oncology, pulmonology, and non-surgical acute admission units, who had a peripheral intravenous catheter (PIVC) or urinary catheter on the days of data collection, were screened for inclusion.

After RICAT-1 had ended, a new personal data protection act was implemented in Europe (General Data Protection Regulation, GDPR).^10^ Therefore, written informed consent was mandatory for inclusion. Consequently, patients who were unable to give informed consent were excluded from the RICAT-2 study. Further, we excluded patients who were admitted for an elective short stay, patients who were terminally ill, and patients with chronic urinary catheters.

#### Outcomes

The primary endpoint was the pooled percentage of PIVCs with an inappropriate indication on the days of data collection, because the use of inappropriate PIVCs was significantly reduced in RICAT-1.

Secondary endpoints included the percentage of inappropriate use of second PIVCs in patients who had more than one PIVC on the days of data collection, and the percentage of patients with urinary catheters with an inappropriate indication on the days of data collection. The last inserted PIVC was regarded as the second PIVC. The appropriate and inappropriate indications were the same as in RICAT-1 (Supplemental Table S1).

#### Data collection

TMZXKvH screened all adult patients with a PIVC and/or a urinary catheter for inclusion in each hospital every other week, which was equal to the methods of the RICAT-1 study. We collected the following data by medical chart review: sex, year of birth, Charlson Comorbidity Index, hospital and ward on inclusion day, admission date, admission in a single isolation room, and admission duration at the intensive care unit if applicable. Further, we collected the number of PIVCs present on the inclusion day, the insertion date, and the clinical indication. All data were collected in Castor Electronic Data Capture.^11^

When the indication for use of the PIVC or urinary catheter was uncertain or could not be found in the medical record, we did not ask the responsible nurse or physician for the indication to prevent interference with the measurement. To assure data validity, a physician of the research team (BJL) audited a random sample of 10% of the data.

#### Statistical analysis

The sample size calculation was based on the primary outcome, the percentage of inappropriate use of PIVCs, since this was reduced significantly in RICAT-1 from 22% to 14%.

For the current study, our hypothesis was that the percentage of inappropriate use of PIVCs would still be less than 15%, five years after RICAT-1, compared to the 22% of the baseline period.^7^ Given the absence of trend (slope change) in the RICAT-1 study and the primary analysis of the RICAT-1 pooled data results, we expected the interrupted time series (ITS) analysis to be equivalent to a before-after comparison. Therefore, we used a power of 80% and an alpha of 0.05 (Supplemental Table S2). When RICAT-2 included at least 307 patients having an overall percentage of 15% or less, power would be at least 80% based on the standard error. We continued our measurements during the study period to perform a supportive ITS analysis.

For the analyses, we used the RICAT-1 baseline data of five of the seven hospitals that also participated in RICAT-2. For comparisons of raw data of the baseline (RICAT-1) and five years after the strategy (RICAT-2), we used logistic regression analysis on data pooled across hospitals and reported odds ratios (ORs) with 95% confidence intervals (95% CI). We used forward selection to identify relevant confounders, i.e., acute admission, admission to intensive care unit (ICU), admission to a single isolation room, Charlson Comorbidity Index ≥ 3, and age, based on previously identified risk factors associated with inappropriate catheter use.^12^ We considered confounders relevant if the regression coefficient changed at least 10% after adding the confounder to the model, which were acute admission and admission to ICU. We used Akaike’s Information Criterion (AIC) and ANOVA tables to compare the fitting of the unadjusted and adjusted models.

As supportive analyses, we conducted an ITS segmented regression analysis to compare the percentages of inappropriate PIVCs between the RICAT-1 baseline and RICAT-2, and the RICAT-1 post-intervention measurement and RICAT-2, respectively. A two-sided p-value of <0.05 was considered significant. We used the Durbin–Watson statistic to test for autocorrelation.

We used IBM SPSS Statistic version 28.0 for the descriptive analyses and R version 4.0.3 (2020-10-10) using the lme4 package for the logistic regression and ITS analyses.

### Qualitative part

#### Participants

We invited 22 clinicians (nurses and physicians) and ward managers who were either involved in the RICAT-1 study and still employed in the same hospital, currently involved in the management of catheter use, or currently involved in quality management of their ward, for interview.

#### Interview guide

We developed two interview guides: for participants that were familiar with RICAT-1, and for participants not familiar with RICAT-1 (Supplemental Table S2). We asked the participants for each of the RICAT-1 strategy components whether these were maintained or currently active in their ward, if they used other strategies regarding appropriate use of catheters, and what factors had hindered or facilitated. We used an existing framework for the sustainability of professional services to ensure that our interview guide contained questions regarding all the potentially relevant factors.^13^

The interviews were performed by EWV and CH via videoconferencing. The qualitative part of the study was performed by researchers who were independent of the RICAT-1 study to prevent bias towards socially desirable responses from the interview participants. The interviews were audio-recorded and informed consent was given.

#### Analysis

The interviews were transcribed and analysed by EWV and CH through inductive thematic analysis in Atlas.ti version 23.1.1. The first three interviews were double-coded independently by both researchers, after which the codes were compared and discussed. The subsequent interviews were alternately coded by one researcher and checked by the other. Through constant comparison and review, codes were merged and rearranged. The emerging barriers and facilitators were fit into the framework for the sustainability of professional services by Crespo-Gonzalez in discussion with the research team.^13^

#### Ethics

On January 5, 2022, the Medical Ethics Review Committee of the Academic Medical Center in Amsterdam confirmed that the Medical Research Involving Human Subjects Act (WMO) did not apply to this study. The study was registered in the ISRCTN registry (https://doi.org/10.1186/ISRCTN18989217). Local approval was obtained in all participating hospitals. We obtained informed consent from all participants for using their data relevant to our research question.

#### Role of funding source

This project was funded by The Netherlands Organisation for Health Research and Development (project number: 839205002). The sponsor had no role in the design and conduct of the study, the collection, management, analysis, and interpretation of the data, the preparation, review, or approval of the manuscript; and decision to submit the manuscript for publication.

## Results

### Quantitative part

#### Study population

**Fig. 1:**
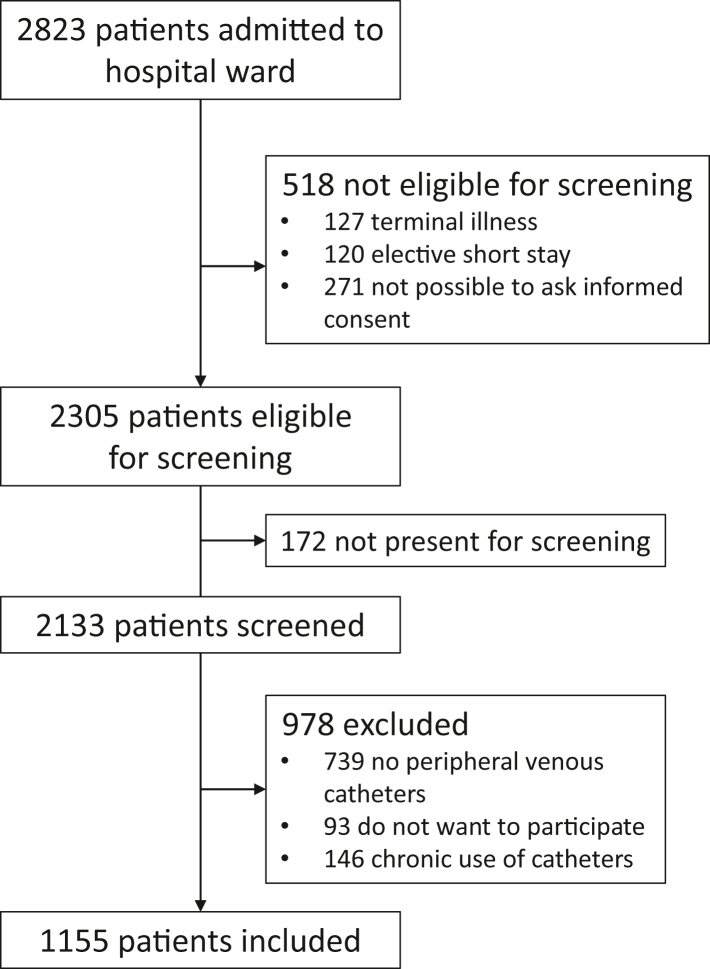
**Flowchart of study inclusion in RICAT-2**.

We included 1179 PIVCs that were present in 1113 unique patients, and 151 patients with a urinary catheter (Fig. 1). After excluding two hospitals from the RICAT-1 baseline data that did not participate in RICAT-2 (Supplemental Table S3), we included 1340 patients for the analyses in this study. We presented the baseline characteristics of the included patients in the RICAT-1 baseline data and RICAT-2 in Table 1.

**Table 1:** Baseline characteristics RICAT-1 baseline & RICAT-2.

Characteristic	RICAT-1 baseline n = 1340	RICAT-2 n = 1155
Male	714 (53.3)	614 (53.2)
Median age [IQR]	67.0 [54.0, 77.0]	67.0 [55.0, 76.5]
Type of catheter during inclusion^a^		
PIVC	1284 (62.5)	1113 (54.6)
Second PIVC	74 (3.6)	66 (3.2)
Urinary catheter	243 (11.8)	151 (7.4)
Median days from admission to inclusion [IQR]	3.0 [2.0, 6.8]	3.0 [1.0, 6.0]
CCI score median [IQR]	2.0 [0.0, 3.0]	1.0 [0.0, 3.0]
CCI ≥ 3	441 (32.9)	326 (28.2)
Acute admission	1198 (89.4)	1087 (94.1)
ICU admission	124 (9.3)	61 (5.3)
ICU days median [IQR]	2.0 [1.0, 6.0]	2.0 [1.0, 5.0]
Isolation	139 (10.4)	147 (12.7)
Hospital		
1.	439 (32.8)	247 (21.4)
2.	162 (12.1)	191 (16.5)
3.	202 (15.1)	232 (20.1)
4.	427 (31.9)	323 (28.0)
5.	110 (8.2)	162 (14.0)
Treating specialty		
Geriatrics	62 (4.6)	21 (1.8)
Internal medicine	563 (42.0)	535 (46.3)
Pulmonology	234 (17.5)	156 (13.5)
Gastroenterology	234 (17.5)	198 (17.1)
Oncology/Haematology	204 (15.2)	164 (14.2)
Other	43 (3.2)	81 (7.0)

IQR: interquartile range; PIVC: peripheral intravenous catheter; CCI: Charlson Comorbidity Index; ICU: Intensive Care Unit.

a Denominators are the patients screened by direct observations.

### Primary outcome

Five years after the de-implementation of inappropriate use of catheters, the percentage of inappropriate PIVCs was still significantly lower than before the strategy (Table 2). Adjusting for confounders (acute admission and admission to ICU) did not lead to a relevant change in the regression coefficient. Therefore, we report the results of the unadjusted model.

**Table 2:** Before-after analyses of inappropriate use of peripheral intravenous catheters and urinary catheters.

	RICAT-1 baseline	RICAT-2	Odds ratio (95% CI)	p value	Adjusted odds ratio (95% CI)	p value
PIVC	282/1284 (22.0%)	154/1113 (13.8%)	0.76 (0.68–0.84)	<0.001	n.a.	n.a.
Second PIVC	30/74 (40.5%)	27/66 (40.9%)	1.01 (0.72–1.41)	0.97	n.a.	n.a.
Urinary catheters	78/243 (32.1%)	55/151 (36.4%)	1.10 (0.89–1.36)	0.38	1.12 (0.89–1.40)	0.22

n.a.: not applicable.

### Supportive analyses

**Fig. 2:**
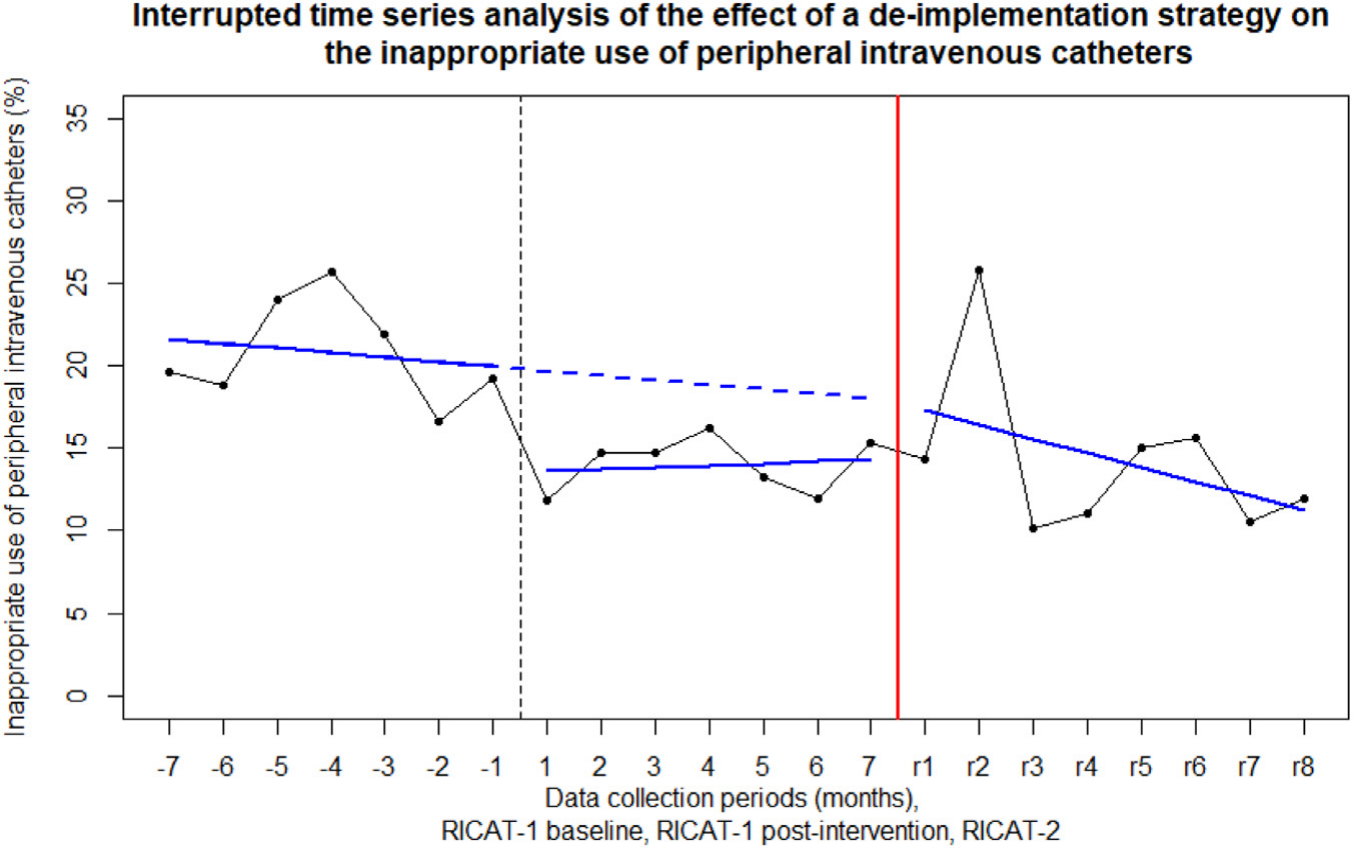
**Interrupted time series analysis of the effect of a de-implementation strategy on the inappropriate use of peripheral intravenous catheters.** The x-axis represents the data collection periods in months within the study periods. RICAT-1 baseline on the left (−7 to −1 on the x-axis), RICAT-1 intervention in the middle (1–7 on the x-axis), RICAT-2 study on the right side (r1 to r8 on the x-axis). The horizontal solid blue lines are the trend lines based on the ITS analysis in the RICAT-1 study and RICAT-2 study. The horizontal dashed line shows the expected trend line in the RICAT-1 intervention period based on the RICAT-1 baseline period without intervention.

The results of the ITS analysis of the RICAT-1 baseline and RICAT-2 data showed no significant sustained reduction of inappropriate use of PIVCs (Fig. 2). The Durbin–Watson statistic was 2.42 (autocorrelation coefficient −0.22, p = 0.93), indicating negative autocorrelation. However, adjusting for autocorrelation did not result in a better fit of the model (AIC adjusted 93.43 versus AIC unadjusted 92.20, p = 0.38). Therefore, we report the results of the unadjusted model. The intercept (B0) was 21.93 (95% CI 13.79 to 30.07, p = 0.00), the slope (B1) −0.28 (95% CI −2.09 to 1.54, p = 0.75), the level change (B2) −1.83 (95% CI −11.79 to 8.13, p = 0.69), and the slope change (B3) −0.59 (95% CI −2.94 to 1.76, p = 0.59). In addition, the ITS analysis between the RICAT-1 post-intervention and RICAT-2 data also showed no significant change (Supplemental Fig. S1 and Table S5).

### Secondary outcomes

We found no significant difference in the inappropriate use of a second inserted PIVCs and urinary catheters before and five years after the strategy (Table 2). To adjust for confounders in the analysis of inappropriate urinary catheters, we included acute admission and admission to the ICU in the model (AIC adjusted 467.74 versus AIC unadjusted 503.08, p < 0.001). The adjusted model also showed no sustained effects on inappropriate use of catheters. The clinical indications for the use of PIVCs and urinary catheters of the RICAT-2 study are shown in Supplemental Table S5.

### Qualitative part

#### Interview and participant characteristics

We performed 13 interviews with a total of 18 participants. Ten interviews were with one participant, two with two participants from the same hospital, and one interview was with four participants from the same hospital. We interviewed three to five people from each of the five hospitals. The participants’ positions were senior nurse, regular nurse, infectious disease specialist (physician or nurse), or team manager. Several participants were also a member of their hospital’s antimicrobial stewardship team. The interviews lasted between 13 and 51 min with a median duration of 21 min. The interviews with participants from hospitals that actively maintained strategy components were significantly longer than those who did not maintain the strategy. If strategy components were maintained, for each component could be questioned why and how this was maintained.

#### Maintenance of strategy components

The maintained activities from hospitals 3 and 5 were coordinated by a dedicated project team (Table 3). The other hospitals did not have a dedicated project team. Some currently active strategy components had been inactive or absent for some time in the last five years and were renewed. Several strategy components existed in different forms, such as education through e-learning instead of meetings or the pocket cards being uploaded in the digital protocol. Hospitals 1 and 3 initiated an extra strategy component: they trained nurses to insert PIVCs themselves to lower the threshold to remove them as they did not depend anymore on doctors to insert PIVCs.

**Table 3:** Effects and maintenance of the original RICAT-1 strategy components.

Percentages of inappropriate PIVCs				Strategy components						
Hospital	RICAT-1 baseline	RICAT-1 Post-intervention	RICAT-2	Audit and feedback on the number of inappropriate catheters.	Educational meetings or e-learnings for physicians and nurses.	Local champion.	Pocket cards, posters, digital protocols with the appropriateness criteria.	Smart phrase in electronic health record (originally in hospital 2 only).	Empowerment for nurses to independently remove catheters (originally in hospital 3 only).	New component after RICAT-1 ended.
1	19.1%	13.5%	13.0%	-	-	=	-	n.a.	n.a.	+
2	25.9%	14.4%	13.4%	-	-	-	-	-	n.a.	n.a.
3	25.0%	14.1%	21.9%	=	=	=	=	+	=	+
4	22.7%	16.0%	11.3%	-	-	-	=	+	+	n.a.
5	18.4%	15.0%	9.0%	=	=	=	=	n.a.	n.a.	n.a.

-: stopped; =: maintained; +: new; n.a. not applicable; PIVCs: peripheral intravenous catheter.

Some strategy components were present but did not fully achieve their effect: for example, some participants reported that the clinical champions were not active or known by colleagues, the wards did nothing with the feedback, and posters and protocols were not noticed. On the contrary, some strategy components were not necessary to achieve the desired effect. Respondents from hospitals 1, 2, and 5 reported that the appropriateness of the patients’ catheters was discussed in daily rounds, even without the smart phrase. Respondents from four hospitals thought that clinicians who were exposed to the strategy during RICAT-1 had permanently changed their way of working with catheters and that the strategy was mainly for the new employees. Opinions on the necessity of maintaining the strategy also differed: some participants felt motivated to further decrease inappropriate catheter use, while others found that decreasing attention to a subject is normal and creates room for new projects.

#### Barriers and facilitators

The participants reported several barriers and facilitators to maintaining or starting strategy components for reducing inappropriate catheters. These are summarized below and fully reported in Supplemental Table S6.

Almost all hospitals experienced barriers related to strategic planning: other projects or COVID-19 care were prioritized over maintaining the RICAT strategy. Combined with a lack of time or personnel, there was little room left to coordinate the strategy. A lack of a (well-informed) clinical champion was also frequently reported. For two hospitals, part of the strategy was stopped when their wards were relocated. Frequently mentioned facilitators included a motivation to reduce the length of stay and to prevent infections. Participants from hospital 5 experienced only facilitators and no barriers, contrary to the other hospitals that experienced both.

## Discussion

We found a sustained reduction in inappropriate use of PIVCs five years after the introduction of a multifaceted de-implementation strategy in five hospitals in the Netherlands. Two of the five hospitals had a dedicated project team and maintained most of the strategy components, while the other three hospitals maintained zero to a few components. The main barriers to maintaining the strategy were prioritization of other projects or COVID-19 care, a lack of time and/or personnel, and a lack of a (well-informed) local champion. The main facilitating factor was the clinicians’ motivation to prevent infections and reduce the length of hospital stay.

Despite the hospitals’ variety of efforts to maintain strategy components, the effect was sustained in most hospitals regarding the primary endpoint. Some hospitals maintained the effect with zero to a few strategy components and no project team, while another hospital kept a smaller effect despite maintaining the strategy. Positively, temporary investment in the de-implementation strategy was in these hospitals sufficient to achieve a long-term behavioural change, because a positive correlation between strategy compliance and reductions in inappropriate use of catheters was observed in RICAT-1.^7^ The current results suggest that achieving an immediate effect requires a different approach than the sustainment of an effect regarding strategy compliance. Since each hospital’s culture, context, and the fine details of the strategy are different, it is impossible to study what exact strategy will work for all hospitals.^14^, ^15^, ^16^ Based on our results, periodically measuring the inappropriate use of catheters in hospital wards may be efficient to provide healthcare organisations with information regarding their performance status to assess whether organizational adjustments are necessary to improve healthcare practices.

Further, between RICAT-1 and RICAT-2, the government of the Netherlands funded two programs that spurred the de-implementation of low-value care, which might have influenced the use of inappropriate catheters. One program supported project teams in hospitals that wanted to reduce the use of inappropriate catheters, in a nationwide network called ‘Better without catheter’,^17^ and the other program spread a list of recommendations amongst all hospitals in the Netherlands, including a recommendation on removing inappropriate catheters.^18^ Nevertheless, only hospital 5 actively participated in this program, suggesting that this program did not influence the maintenance of the effect in the primary before-after analysis.

Contrary to the before-after analysis, the ITS analyses showed no significant sustained reduction of inappropriate PIVCs. A possible explanation is the small number of included patients (n < 100) and a high percentage of inappropriate use in the second month, leading to a relatively high percentage of inappropriate PIVC use, causing a negative time trend (Fig. 2).^19^ Further, the absolute numbers of inappropriate urinary catheters were similar in RICAT-2 compared to the RICAT-1 intervention measurement, but the number of included catheters (denominators) was lower, leading to a relatively high percentage of inappropriate use. These results confirm that the de-implementation strategy in RICAT-1 focused on the timely removal of catheters, rather than on the clinical indications for inserting them in the first place. When looking at the inappropriate indications for urinary catheter use in RICAT-2, a relatively large part was used for ill and fatigued patients who had been given a urinary catheter for comfort reasons. While this is not a medical indication for a urinary catheter, it might be right in terms of individualized patient care.

A major strength of our study is that we evaluated the long-term effects five years after the de-implementation strategy had been rolled out, whereas the few studies that investigated long-term effects evaluated a shorter follow-up period immediately after the implementation of a strategy.^20^^,^^21^ Further, by using a mixed-methods design, we could further elaborate and clarify the quantitative findings. In addition, we collected the quantitative data in the background without clinicians being aware of the measurement, and the interviews were performed by researchers who were not known by the hospitals as the RICAT-1 researchers.

Some limitations of our study should be taken into consideration. Firstly, we excluded two of seven hospitals in the RICAT-2 study due to feasibility reasons, mainly due to organizational changes that caused some wards to disappear due to mergers with other wards. Regarding the included hospitals, we did not find destruction of the benefits of the de-implementation strategy. Therefore, we believe that organizational and structural changes do not necessarily worsen the sustained effect of the de-implementation strategy. Still, the five included hospitals still varied in terms of size, academic, and general teaching, providing generalisability. Secondly, our study lacked appropriate numbers to perform a non-inferiority analysis between the RICAT-1 post-intervention and the RICAT-2 data. An additional ITS analysis between these study periods did not show a significant change in inappropriate use of PIVCs. However, it is not possible to draw firm conclusions about non-inferiority from ITS analysis. Thirdly, although the demographic data in both studies are comparable, we could not include severely ill patients due to privacy legislation. However, we assumed that severely ill patients had appropriate indications for catheters. Therefore, we believe the current results do not underestimate the inappropriate use and can be generalized to the full study population of internal medicine and non-surgical subspecialty wards. Fourthly, despite our efforts to invite more frontline nurses to participate in our interviews, we were unable to include them. Finally, despite only informing one person per hospital about this follow-up measurement, we cannot guarantee that the Hawthorne effect did not influence the results.

Inappropriate catheter use was still significantly lower than before the strategy, suggesting a sustained effect five years after the implementation of a multifaceted de-implementation strategy on the appropriate use of PIVCs in five hospitals. We observed no association between the number and mix of strategy components that hospitals maintained and the sustainment of the effect. For some hospitals, a temporary investment in this de-implementation strategy might be sufficient to achieve sustained effects. We recommend other hospitals adopt this de-implementation strategy to reduce inappropriate catheter use.

## Contributors

Conceptualization: SEG, RBK, BJL. Methodology: TMZXKvH, EWV, BJL. Software: n.a. Validation: TMZXKvH, EWV, BJL. Formal analysis: TMZXKvH, EWV, BJL. Investigation: TMZXKvH, EWV. Resources: TMZXKvH, EWV. Data curation: TMZXKvH, EWV. Writing—Original Draft: TMZXKvH, EWV. Writing—Review & Editing: SEG, RBK, BJL. Visualization: TMZXKvH, EWV. Supervision: SEG, BJL. Project Administration: TMZXKvH, EWV. Funding acquisition: SEG, RBK, BJL. All authors have read and approved the final version of the manuscript. TMZXKvH, EV, and BL had full access to all the data in the study and take responsibility for the integrity of the data and the accuracy of the data analysis.

## Data sharing statement

The de-identified data are available upon publication to investigators whose proposed use of the data has been approved by an independent review committee identified for this purpose. Proposals should be directed to the principal investigator Prof. Dr. Suzanne E. Geerlings (s.e.geerlings@amsterdamumc.nl). To gain access, data requestors will need to sign a data access agreement. The interview data is not available due to the sensitive nature of the data.

## Declaration of interests

BJL received honoraria for giving a lecture at Coloplast BV. SEG received funding from the Netherlands Organisation for Healthcare research and Development for this project, and a consulting fee from Immunotek as a member of the advisory board about a vaccine to prevent urinary tract infections which was paid to the affiliated institution. The other authors declare no conflicts of interests.
