# Supplementary material for: Five-year sustainability of a de-implementation strategy to reduce inappropriate use of catheters: a multicentre, mixed-methods study
Source: eClinicalMedicine. 2024 Aug 16;75:102785. doi: 10.1016/j.eclinm.2024.102785 (PMC11701439; doi:10.1016/j.eclinm.2024.102785)
Supplement: Supplemental Fig. S1 and Tables S1–S6 [file mmc1.docx]

**Supplementary files belonging to Five-year sustainability of a de-implementation strategy to reduce inappropriate use of catheters**

**Supplemental table 1. Appropriate indications for catheter use**

| **Peripheral intravenous catheter** |
| --- |
| - Delivery of peripherally compatible infusate (intravenous fluids and medications) at least once in 24 hours. - Injection of contrast fluids - Intravenous access for cardiac dysrhythmia - Transfusion of blood and blood products |
| **Urinary** **catheter** |
| - Accurate measurement of urinary output in patients who are critically ill when required for treatment - Acute urinary retention or bladder outlet obstruction (≥150 mL) - Assist in healing or open sacral or perineal wounds in patients with urinary incontinence - Continuous bladder irrigation for haematuria - Palliative care for patients who are terminally ill if needed - Patient requires prolonged immobilization - Before or after surgery according to (local) protocol - Volume measurements of urine output for diagnostics (24h urine) that cannot be assessed by other collection strategies |

**Supplemental table 2. Topic guide for interviews in the RICAT-2 study**

**Interview participant familiar with Ricat-1**

- What was your role during Ricat-1?
- What did you think of the effect at the end of Ricat-1?
- Did you communicate about this effect in your hospital or ward? What were the responses?
- What happened directly after Ricat-1, was there for example a discussion on keeping the strategy? If yes, how was this discussion? If no, how come?
- Was there attention to appropriate catheter use after Ricat-1?
- For every strategy component:
  - Is this strategy component continued?
    - Feedback
    - Educational meetings
    - Local champion and a meeting for the local champions
    - Pocket cards and posters
    - Smartphrase
    - Empowered nurses
  - If yes, in what form? How did it go? Did you run into any challenges? How did you deal with those?
  - If no, what is the reason that is was not continued? Who were involved in this?
- What did you do to maintain the strategy? Were there any challenging factors? Or any facilitating?
- Did anyone set goals or a plan to maintain the strategy or the results? Why/why not?
- Is appropriate catheter use in the training program for new employees?
- Are the appropriate and inappropriate indications in the protocols?
- Are there any new actions for appropriate catheter use since Ricat-1?
- Were there any developments that influenced maintaining the results? Where there any actions that influenced the appropriate use of catheters on your ward/in your hospital?
- What did you miss? What would have helped?

**Interview participant not familiar with Ricat-1**

- From 2016-2018 there was a study that reduced inappropriate use of urinary and intravasal catheters. Are there any activities for appropriate catheter use now on your ward/in your hospital? Who on your ward is involved in this?
- For every strategy component:
  - Is this component currently active? In what form?
    - Feedback
    - Educational meetings
    - Local champion and a meeting for the local champions
    - Pocket cards and posters
    - Smartphrase
    - Empowered nurses
  - When did it start and with what cause/motivation?
  - How did it go? Did you run into any challenges? How did you deal with those?
- Were there any developments that influenced maintaining the results? Where there any actions that influenced the appropriate use of catheters on your ward/in your hospital?
- Is appropriate catheter use in the training program for new employees?
- Are the appropriate and inappropriate indications in the protocols?
- What did you miss? What would have helped?

**Supplemental table 3. Percentages of inappropriate use of peripheral intravenous catheters and urinary catheters per hospital**

| Hospital | RICAT-1 baseline | RICAT-1 intervention | RICAT-2 long-term* |
| --- | --- | --- | --- |
| ***PIVCs*** |  |  |  |
| 1 | 79/413 (19·1%) | 60/445 (13·5%) | 31/238 (13·0%) |
| 2 | 41/158 (25·9%) | 30/209 (14·4%) | 25/186 (13·4%) |
| 3 | 49/196 (25·0%) | 36/255 (14·1%) | 49/224 (21·9%) |
| 4 | 94/414 (22·7%) | 66/413 (16·0%) | 35/310 (11·3%) |
| 5 | 19/103 (18·4%) | 32/213 (15·0%) | 14/155 (9·0%) |
| Total | 282/1284 (22·0%) | 224/1535 (14·6%) | 154/1113 (13·8%) |
| ***Urinary catheters*** |  |  |  |
| 1 | 27/98 (27·6%) | 8/83 (9·6%) | 6/39 (15·4%) |
| 2 | 11/23 (47·8%) | 11/48 (22·9%) | 10/21 (47·6%) |
| 3 | 16/41 (39·0%) | 16/54 (29·6%) | 13/25 (52·0%) |
| 4 | 19/62 (30·6%) | 20/62 (32·3%) | 15/45 (33·3%) |
| 5 | 5/19 (26·3%) | 11/44 (25·0%) | 11/21 (52·4%) |
| Total | 105/324 (32·4%) | 66/291 (22·7%) | 55/151 (36·4%) |

*The RICAT-2 measurement was performed five years after the RICAT-1 baseline period

PIVCs: peripheral intravenous catheters

**Supplemental table 4. Results of secondary interrupted time series analysis between RICAT-1 post-intervention measurement and RICAT-2**

|  | Coefficient | Std. Error | 95% CI | P waarde |
| --- | --- | --- | --- | --- |
| Intercept (B0) | 13.49 | 3.31 | 6.20-20.77 | 0.002 |
| Trend baseline (B1) | 0.12 | 0.74 | -1.51 – 1.75 | 0.87 |
| Level change (B2) | 3.84 | 4.05 | -5.08 – 12.76 | 0.36 |
| Trend difference (B3) | -0.99 | 0.96 | -3.09-1.11 | 0.32 |

**Supplementary figure 1. Secondary interrupted time series analysis between RICAT-1 post-intervention measurement and RICAT-2**


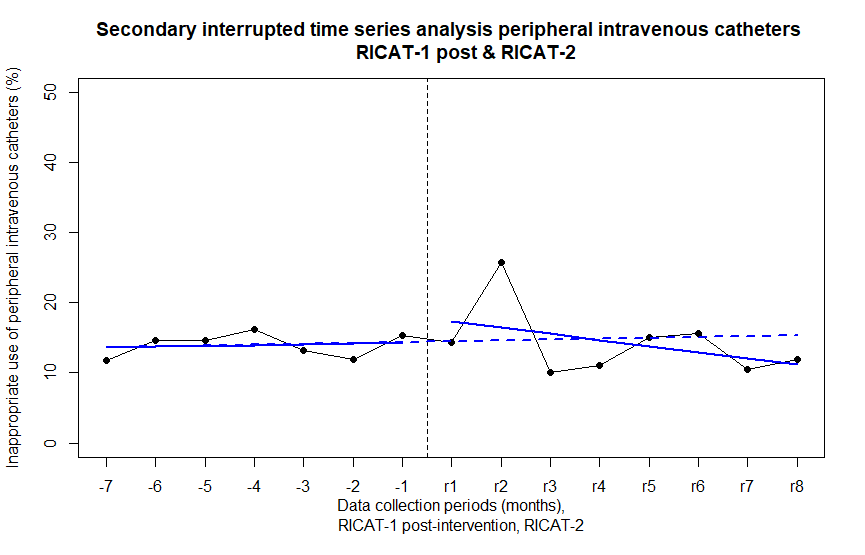


The x-axis represents the data collection periods in months within the study periods. RICAT-1 post-intervention data on the left (-7 to -1 on the x-axis), and RICAT-2 study on the right side (r1 to r8 on the x-axis). The horizontal solid blue lines are the trend lines based on the ITS analysis in the RICAT-1 study and RICAT-2 study. The horizontal dashed line shows the expected trend line in the RICAT-2 study, based on the RICAT-1 post-intervention data.

**Supplemental table 5. Observed clinical indications for catheter use**

| Peripheral intravenous catheters (PIVCs) | 1113 |
| --- | --- |
| Appropriate indication | **959 (86·2%)** |
| *IV fluid and/or medication* | 783 (70·4%) |
| *Transfusion of blood and/or blood products* | 45 (4·0%) |
| *Injection of contrast fluid* | 35 (3·1%) |
| *Other* | 96 (8·6%) |
| Expired appropriate indication | **108 (9·7%)** |
| *IV fluid and/or medication* | 79 (7·1%) |
| *Transfusion of blood and/or blood products* | 5 (0·4%) |
| *Injection of contrast fluid* | 14 (1·3%) |
| *Other* | 10 (0·9%) |
| Inappropriate indication | **33 (3·0%)** |
| *IV therapy, oral intake possible* | 16 (1·4%) |
| *Other* | 17 (1·5%) |
| No or unknown indication | **30 (2·7%)** |
| Urinary catheters | **151** |
| Appropriate indication | **96 (63·6%)** |
| *Urinary retention* | 73 (48·3%) |
| *Measurement urinary output in critically ill patients* | 20 (13·2%) |
| *Palliative care for terminally ill* | 2 (1·3%) |
| *Open sacral or perineal wounds and urinary incontinence* | 1 (0·7%) |
| Expired appropriate indication | **27 (17·9%)** |
| *Measurement urinary output in critically ill patients* | 20 (13·2%) |
| *Other* | 7 (4·6%) |
| Inappropriate indication | **28 (18·5%)** |
| *Urinary incontinence* | 2 (1·3%) |
| *Intravenous diuretics* | 5 (3·3%) |
| *Patient request* | 1 (0·7%) |
| *Measurement urinary output in non-critically ill patients* | 7 (4·6%) |
| *Other* | 13 (8·6%) |
| Second PIVCs | **66** |
| Appropriate indication | 22 (33·3%) |
| Expired appropriate indication | 15 (22·7%) |
| Inappropriate indication | 2 (3·0%) |
| No or unknown indication | 10 (15·2%) |

IV: intravenous. PIVC: peripheral intravenous catheters

**Supplemental table 6. Barriers and facilitators to the de-implementation strategy**

|  |  | **Framework category** | **Barrier*** | **Facilitator*** |
| --- | --- | --- | --- | --- |
| **External context/ environment** | Socioeconomic and sociopolitical environment | Political support/policies/regulations/legislations | Project is not imposed by the hospital’s management (4) | The ward’s priorities align with the project (4) |
|  |  | Enabling environment/access to care | Relocation of the ward (1, 2) |  |
| **Local environment** | Partners/stakeholders | Networking/involvement/participations/engagement/commitment |  | Support of a national network Better without catheter (3) |
| **Organization** | Staff | Skills evaluation/monitoring | Complications/infections are not monitored (3) |  |
|  |  | Motivation/involvement/trust/goals/strategic planning | Subject is not prioritized because of many other projects (1, 2, 4)  Covid-19-care was prioritized (1, 2, 3, 4) | Motivation to reduce length of stay (2, 4)  Motivated when an infection occurs (2, 3) |
|  | Innovation provision | Knowledge/information | Many other pocket cards and posters are present (2) |  |
|  |  | Workload/staffing | High turnover of personnel (1, 2, 3, 4)  Personnel shortage (2)  No administrative support (3) |  |
|  | Leader/champion | Organizational capacity/governance | Unclear who should take responsibility (1, 3) |  |
|  |  | Leadership | Departure of the researcher/clinical champion, no transfer to new/other employee (1, 3, 4) | Physician initiated the project and appointed a team of nurses (5)  The team is highly motivated (5) |
| **Innovation** | Process | Program evaluation/monitoring/data evaluation |  | Data from multiple departments motivates (5) |
|  |  | Integration/fit/alignment/compatibility |  | Subject was already addressed in protocols (4) |
|  | Characteristics | Benefits/effectiveness/legitimacy/quality |  | Project reduces catheter-associated infections (1, 2, 3) |
|  | Resources | Time | Lack of time, intensity of care per patient increases (2, 3) |  |

*the number indicates the hospital in which the factor was present
